# Relationship between knee isokinetic muscle strength and countermovement jump height among elite male gymnasts

**DOI:** 10.3389/fspor.2025.1627059

**Published:** 2025-09-01

**Authors:** Yijie Ma, Zixiang Zhou, Rangxi Jin, Dexin Wang, Chao Chen

**Affiliations:** ^1^ School of Athletic Performance, Shanghai University of Sport, Shanghai, China; ^2^ Physical Education, Ludong University School, Shandong, China; ^3^ College of Physical Education, Dalian University, Dalian, Liaoning, China

**Keywords:** muscle strength, muscle balance, vertical jump, hamstring to quadricepsratio, limb asymmetries

## Abstract

**Introduction:**

This study quantifies the isokinetic knee strength of elite male gymnasts and examines the relationship between concentric extensor strength, limb asymmetry, and countermovement jump (CMJ) height.

**Methods:**

A total of 18 elite male gymnasts participated in isokinetic strength testing, assessing concentric and eccentric actions during both extension and flexion of the knee at joint angular velocities of 60°/s, 180°/s, and 240°/s. Relative peak torque (RPT) of the knee flexors and extensors, the functional eccentric hamstring/concentric quadriceps (H_ecc_/Q_con_) ratio, and the inter-limb asymmetries were analyzed. CMJ height was assessed using a contact mat, and the relationship between concentric extensor RPT and limb asymmetries with CMJ height was analyzed using Pearson correlation coefficients analysis.

**Results:**

At an angular velocity of 180°/s, both an abnormal limb asymmetries in the concentric extensors was observed and the strongest negative correlation with CMJ height (*r* = −0.638, *p* < 0.05) was found. At an angular velocity of 240°/s, knee extensor strength demonstrated the strongest correlation with CMJ height (*r* = 0.962, *P* < 0.001).

**Discussion:**

Therefore, we recommend that gymnasts conduct regular isokinetic strength assessments, particularly focusing on concentric knee extensor strength and limb asymmetries, as they exhibit moderate to high correlations with CMJ height.

## Introduction

Gymnast scores are determined by the difficulty and execution quality of their movements (1). According to Fédération Internationale de Gymnastique rules, the height and quality of a somersault can impact gymnast scores. Somersault height depends on the take-off quality, while air time is determined by the vertical velocity of the take-off (2). Some researchers have highlighted the importance of lower limb strength for jumping and landing among elite gymnasts (3, 4). However, insufficient lower limb strength and muscle imbalances may present potential injury risks (5–7). Specifically, the strength of the knee flexor and extensor muscles significantly impacts take-off and landing in somersaults and thereby affect gymnasts’ performance (8, 9).

Vault and tumbling routines in gymnastics require rapid force production in jumping and running tasks (10). A longer airborne duration grants athletes more time to show aerial postures and complete mid-air maneuvers (11), while an optimal amount of leg volume and mass contributes to the success of gymnasts (12). The stretch-shortening cycle allows muscles to store elastic potential energy during a rapid eccentric (lengthening) phase and subsequently release it during the concentric (contracting) phase, resulting in increased force output. Countermovement jumps (CMJ) are commonly used to assess this ability in the lower extremities (13). This movement involves flexion at the hips and knees initially, followed by rapid extension prior to the jump itself, characteristics that line well with the take-off characteristics of tumbling movements (14). Extensor strength has been shown to significantly correlate with jump height across different sports, such as volleyball [*r* = 0.479; (15)] and basketball [*r* = 0.88; (16)], highlighting its critical role in jumping-related sports.

Inter-limb asymmetry, defined as the percentage difference in muscle strength or performance output between the dominant and non-dominant legs (17), has long been a topical factor in research and monitoring, due to its potential impacts on athletic performance. Its impact may vary across sports (18), with strength asymmetries observed in disciplines like sprinting (19), swimming (20) and basketball (21). Research suggests that an asymmetry of approximately 10% can reduce jump height and prolong change-of-direction time (22, 23). Furthermore, limb asymmetry exceeding 15% may be associated with an increased injury risk in athletes (17), implying that minimizing asymmetry may benefit performance. However, the use of, and the relevancy of 10% or 15% thresholds, is disputed within the sport performance and rehabilitation fields. Contradictory findings exist in relation to asymmetry and performance, with some studies reporting no impact of inter-limb asymmetries on athletes’ sprint and change-of-direction performance (24, 25). These discrepancies suggest the crucial role of sport specificity, as different sports require distinct abilities. Therefore, further research is vital to elucidate the relationship between Inter-limb asymmetry and jumping performance, particularly in gymnasts.

The contraction of the agonist muscle is often accompanied by the activation of the antagonist. The functional H/Q ratio is defined as the ratio of eccentric knee flexor strength to concentric knee extensor strength, reflecting the synergistic action between the quadriceps and hamstrings during knee movement. Kyselovičová et al. (26) reported that knee muscular strength can influence the dynamic balancing ability of gymnasts. Additionally, a study by Mandroukas et al. (27) found that the isokinetic strength of the lower extremity extensors was significantly greater in pre-pubescent female gymnasts compared to an age-matched control group, a difference which was not observed in the flexors. Numerous other studies suggest that maintaining a high H/Q ratio in the knee joint can positively contribute to injury prevention, athletic performance, and post-injury rehabilitation (28, 29).

Although researchers have established a link between knee isokinetic muscle strength and jump performance (15), the relationship between isokinetic knee flexor and extensor strength and athletic performance among elite gymnasts has attracted limited research attention. While previous studies have explored the differences in isokinetic knee strength and dynamic balance among elite female gymnasts from different gymnastic disciplines (rhythmic, artistic, and aerobic gymnastics) (26), as well as differences in lower limb isokinetic strength and joint range of motion between pre-pubescent female gymnasts and age-matched controls (27). However, no researchers have specifically focused on the relationship between lower limb isokinetic muscle strength and CMJ height among elite male gymnasts. As such, the primary aim of this study is to investigate the isokinetic knee strength characteristics of elite gymnasts and to explore the relationship between extensor strength, limb asymmetry, and CMJ height. We hypothesize that isokinetic strength will correlate with CMJ height. Additionally, our secondary aim is to determine how much isokinetic strength explains variations in CMJ height.

## Methods

### Experimental design

A cross-sectional correlational design was used to examine the isokinetic knee strength characteristics of elite gymnasts and their relationship with extensor strength, limb asymmetry, and CMJ height. The concentric and eccentric peak torque of knee flexors and extensors in gymnasts was measured using an isokinetic dynamometer (Biodex Medical Systems IV; Shirley, NY, USA) to analyze the variations in knee muscle strength at different velocities (60°/s, 180°/s, and 240°/s). The RPT of the concentric and eccentric knee flexors and extensors was used for further analysis of the functional H_ecc/_Q ratio_con_ and limb asymmetry {limb asymmetry = [(RPT_D_−RPT_ND_)/RPT_D_] ⋅ 100; (15)}. The body weight of each gymnast was measured using a scale for subsequent weight normalization. CMJ height was calculated using the flight time method provided by a contact mat (Smart Jump; Fusion Sport, Coopers Plains, Australia). Relationships between RPT of concentric knee extensors and limb asymmetry with CMJ height was explored. The testing was conducted in two phases. The first phase involved the CMJ assessments, whilst the second phase involved the isokinetic strength test. Both testing sessions were conducted at the same time of day, and participants were asked to ensure they were well hydrated, and to refrain from ingestion of food and caffeine for 3 h prior to the assessments.

### Participants

Using G-Power 3.1 to estimate the sample size for this study, an *F*-test model was applied. A moderate effect size of 0.25 was predicted, with a statistical power (1-β) of 0.8 and a significance level of 0.05. The analysis included two groups and three repeated measures. The calculated sample size was 28. However, the total sample size in this study was limited to 18 participants due to the strict inclusion criterion that required all participants to be elite gymnasts. A total of 18 elite male gymnasts (age = 24.5 ± 3.22 years; height = 162.61 ± 4.68 cm; weight = 57.65 ± 4.17 kg; training years = 19.33 ± 3.54 years) voluntarily participated in this study. As inclusion criteria, all participants were required to engage in three to five hours of training per day, with each participant having competed in at least one international competition and two national championship events. Meanwhile, those participants with a history of lower limb surgery within the past year and any lower limb injury within the eight weeks prior to testing were excluded. All participants completed the Waterloo Footedness Questionnaire (30) to determine their dominant and non-dominant legs. They also signed an informed consent prior to the test, indicating their voluntary participation in the study. This study was approved by the Research Ethics Committee in accordance with the Helsinki Declaration (Approval No: 102772021RT031).

### Testing procedure

#### Lower limb countermovement jump (CMJ) test

The CMJ test was conducted using a contact mat (Smart Jump; Fusion Sport, Coopers Plains, Australia), whose high relative reliability has been confirmed in previous studies (ICC = 0.94) (31). This contact platform provides the flight time of the participants in milliseconds, with the following calculation used to identify jump height (h = 9.81*t^2^/8). Before testing, the participants underwent a 15-minute warm-up session, which included a 7-minute slow jog on a treadmill at 8 kilometers per hour, 8 minutes of foam rolling, and dynamic stretching. They also familiarized themselves with the testing procedure and movements. While jumping, the participants were instructed to place their hands on their hips. When measuring CMJ, the participants started in a standing position and then executed a downward movement followed by a rapid full extension of their lower limbs. To avoid changes in the jumping coordination patterns, the participants were allowed to freely choose the amplitude of their countermovement (32). Each participant performed 5 CMJ attempts, with a 30 s rest interval between each attempt, and they were encouraged to jump as high as possible (33). All participants performed the tests wearing gymnastics shoes, with their best performance was used for the subsequent analysis.

#### Knee isokinetic muscle strength test

The Biodex dynamometer (Biodex Medical Systems IV; Shirley, NY, USA) was used to assess the strength of knee extension and flexion. Before the test, the participants completed a 15 min warm-up session, including 8 min of resistance cycling and 7 min of dynamic stretching. They were then familiarized with the test using different target angular speeds, which was followed by a 2 min rest before the test officially began. Their body, waist, and thighs were secured with stabilizing straps, and their hip and thighs were positioned at a 90° angle. The rotation axis of the machine was aligned with the lateral femoral epicondyle of the knee, and the foot pad was placed 2–3 cm superior to the medial malleolus. Gravity correction was performed before each test. The strength testing of the knee flexors and extensors was conducted in a seated position, with maximum voluntary flexion and extension performed within a knee joint movement range of 10°–110° (with 0° indicating full knee extension). The testing protocol included angular velocities of 60°/s, 180°/s, and 240°/s. To minimize potential order effects, we randomly determined each participant's starting test leg (dominant or non-dominant). Concurrently, the starting angular velocity was randomized, with subsequent testing then proceeding in a fixed sequence (e.g., 180°/s followed by 240°/s and then 60°/s if starting at 180°/s). The strength testing for the knee flexors and extensors was performed 5 repetitions at an angular velocity of 60°/s, 10 repetitions at 180°/s, and 15 repetitions at 240°/s. The maximum peak torque observed throughout these repetitions for each speed was recorded. All knee flexion and extension tests were performed in a concentric and eccentric mode. The participants were allowed to take a one-minute rest between each angular velocity and a three-minute rest between each leg. Before testing, the participants were informed to exert their maximum effort in each test, and during the testing, they were verbally encouraged and motivated to perform to the best of their abilities (34). After testing, the absolute peak torque values were recorded and normalized based on body weight. The relative peak torque was used for the knee extensors and flexors, knee H/Q ratio, and limb asymmetry analyses.

## Statistical analyses

Statistical analyses were conducted using SPSS 26.0 (IBM Corp. Amork, NY). All dependent variables were calculated using descriptive statistics (mean ± standard deviation) and reported with 95% confidence intervals (CI) and coefficients of variation (CV%) to indicate precision (35). The normality of all data was confirmed through the Shapiro Wilk normality test. Subsequently, a two-way (2 × 3) repeated measures ANOVA (leg: dominant, non-dominant; velocity: 60°/s, 180°/s, and 240°/s) was conducted to analyze the RPT of knee flexors and extensors and the functional H/Q ratio. *post-hoc* analysis using the Bonferroni test was then conducted to analyze changes in RPT and functional H/Q ratio at different speeds, with Hedge's *g* effect sizes reported. The Hedge's g effect size is classified into three levels: small = 0.2–0.49, medium = 0.5–0.79, and large ≥0.8 (8). The effect size for the ANOVA was calculated using partial eta squared (*η*p^2^), which was categorized as follows: small *η*p^2^ = 0.01; medium *η*p^2^ = 0.06; and large *η*p^2^ = 0.14. The Pearson product–moment correlation coefficient (r) and simple linear regression analysis were then applied to assess the relationship between RPT (dependent variable) and CMJ (independent variable), and between limb asymmetry (dependent variable) and CMJ (independent variable). The following thresholds were used to qualitatively assess the correlations: <0.1 = trivial; 0.1–0.3 = small; 0.31–0.5 = moderate; 0.51–0.7 = large; 0.71–0.9 = very large; >0.91 = nearly perfect (36). Statistical significance was set at *P* < 0.05.

## Results

### Peak torque

The RPT of the knee joints of 18 gymnasts is shown in Table 1. The interaction effect between leg × angular velocity is not significant for the peak torque of concentric and eccentric knee flexor muscles (F = 0.302, *p* > 0.05, *η*p^2^ = 0.18; F = 0.865, *p* > 0.05, *η*p^2^ = 0.05) or main effect for leg were observed, but there was a significant main effect for angular velocity (F = 67.394, *p* < 0.001, *η*p^2^ = 0.803; F = 5.917, *p* = 0.006, *η*p^2^ = 0.264). For the peak torque of concentric and eccentric knee extensors, no significant leg × angular velocity interactions (F = 0.894, *p* > 0.05, *η*p^2^ = 0.026; F = 0.587, *p* > 0.05, *η*p2 = 0.034) were observed, but there was a main effect for angular velocity (F = 131.683, *p* < 0.001, *η*p^2^ = 0.795; F = 65.227, *p* < 0.001, *η*p2 = 0.798). Bonferroni analysis showed that when the angular velocity increased from 60°/s to 240°/s, the relative PT values of both the concentric knee flexors (dominant: *p* < 0.001, g = 0.489, g = 373; non-dominant: *p* < 0.001, g = 0.476, g = 0.500) and extensors(dominant: *p* < 0.001, g = 1.420, g = 1.011; non-dominant: *p* < 0.001, g = 1.581; *p* < 0.005, g = 0.666) significantly decreased. However, this trend was observed only in the eccentric knee flexors of dominant leg (*p* < 0.05, g = 0.347) when the angular velocity increased from 60°/s to 180°/s, while in the extensors, the same trend was observed when the angular velocity increased from 60°/s to 240°/s (dominant: *p* < 0.001, g = 0.594, *p* < 0.05, g = 0.262; non-dominant: *p* < 0.05, g = 0.564; *p* < 0.001, g = 0.449).

**Table 1:** Relative peak torque in the dominant and non-dominant leg at three angular velocities.

Velocity	60°/s (Nm/kg) 95%CI	CV%	180 /s (Nm/kg) 95%CI	CV%	240°/s (Nm/kg) 95%CI	CV%
CF-D	1.83 ± 0.22 1.72–1.94	12.16	1.73 ± 0.20** 1.62–1.82	11.50	1.65 ± 0.19** 1.55–1.74	11.56
CF-ND	1.73 ± 0.23 1.67–1.84	13.16	1.63 ± 0.18** 1.53–1.72	11.19	1.54 ± 0.16** 1.46–1.62	10.37
CE-D	3.27 ± 0.27 3.12–3.40	8.38	2.81 ± 0.35** 2.63–2.98	12.56	2.34 ± 0.54** 2.06–2.60	23.14
CE-ND	3.12 ± 0.30 2.96–3.26	9.61	2.52 ± 0.42** 2.31–2.73	16.78	2.18 ± 0.56* 1.90–2.46	25.82
EF-D	2.84 ± 0.34 2.66–3.00	12.05	2.71 ± 0.36* 2.53–2.88	13.14	2.66 ± 0.41 2.45–2.86	15.48
EF-ND	2.71 ± 0.33 2.55–2.87	12.11	2.66 ± 0.31 2.50–2.81	11.49	2.61 ± 0.31 2.45–2.76	11.96
EE-D	3.64 ± 0.22 3.53–3.75	6.11	3.45 ± 0.39** 3.26–3.64	11.15	3.36 ± 0.35* 3.18–3.52	10.33
EE-ND	3.51 ± 0.25 3.39–3.63	7.01	3.36 ± 0.29* 3.25–3.50	8.78	3.22 ± 0.30** 3.07–3.36	9.33
CMJ (cm)	61.33 ± 4.73 58.98–63.68	7.70				

CF, concentric flexor; CE, concentric extensor; EF, eccentric flexor; EE, eccentric extensor; D, dominant-leg; ND, non-dominant-leg; CMJ, countermovement jump.

Significant difference between pairs of consecutive angular velocities. *(*P* < 0.05): **(*P* < 0.001).

Data are presented as mean ± standard deviation.

### H/Q ratio

Table 2 results show that there was no significant interaction between leg and angular velocity for the H/Q ratio (F = 2.502, *p* > 0.05, *η*p^2^ = 0.132), but there was a main effect for angular velocity (F = 38.397, *p* < 0.001, *η*p^2^ = 0.699). The *post hoc* Bonferroni test showed that the H/Q ratio increased significantly (dominant: *p* < 0.05, g = 1.030, *p* < 0.001, g = 1.035; non-dominant: *p* < 0.001, g = 1.441, *p* < 0.001, g = 0.776) when the speed increased from 60°/s to 240°/s.

**Table 2:** Functional hamstring to quadriceps ratio (H_ecc_/Q_con_) according to leg and angular velocity variables.

Velocity	D	95%CI	ND	95%CI
60°/s	0.87 ± 0.12	0.8153 0.9301	0.88 ± 0.13	0.8141 0.9394
180°/s	0.98 ± 0.16*	0.8994 1.0570	1.08 ± 0.20**	0.9805 1.1761
240°/s	1.18 ± 0.25**	1.0545 1.3049	1.26 ± 0.32**	1.0991 1.4132

D, dominant; ND, non-dominant.

Significant difference between pairs of consecutive angular velocities. *(*P* < 0.05); **(*P* < 0.001).

Data are presented as mean ± standard deviation.

### Limb asymmetry

The results of the limb asymmetry are shown in Table 3. The limb asymmetry in eccentric knee flexors and extensors, as well as in concentric knee flexors, were all below 10% at the three angular velocities (60°/s, 180°/s, and 240°/s). However, this trend was only observed in concentric knee extensors at angular velocities of 60°/s and 240°/s (4.60% and 6.94%, respectively). At an angular velocity of 180°/s, the knee extensors exhibited a limb asymmetry of 10.02%.

**Table 3:** The limb asymmetry of the dominant and non-dominant legs for both flexors and extensors.

Velocity	60°/s (Nm/kg)	95%CI	180 /s (Nm/kg)	95%CI	240°/s (Nm/kg)	95%CI
CF	5.60 ± 3.00	0.0411–0.0709	5.45 ± 4.16	0.0338–0.0752	6.30 ± 0.07	0.0320–0.0940
CE	4.60 ± 1.70	0.0375–0.0544	10.02 ± 9.63	0.0523–0.1481	6.94 ± 3.59	0.0516 0.0873
EF	4.26 ± 2.79	0.0288–0.0565	1.51 ± 7.17	−0.0206 to 0.0507	1.16 ± 11.73	−0.0468 to 0.0699
EE	3.66 ± 1.38	0.0297–0.0435	2.38 ± 6.38	−0.0079 to 0.0555	3.87 ± 3.85	0.0195–0.0579

CF, concentric flexors; CE, concentric extensors; EF, eccentric flexors; EE, eccentric extensors.

Data are presented as mean ± standard deviation.

### Relationships between the countermovement jump and relative peak torque

The results of Table 4 showed significant (*p* < 0.05) positive correlations between concentric extensors and CMJ height at all velocities. The dominant leg's concentric knee extensors at a velocity of 240°/s had the strongest correlation with CMJ height among the RPT. A significant negative correlation (*p* < 0.05) in knee limb asymmetry was observed only in the concentric flexors at 60°/s and in the concentric extensors at 60°/s, 180°/s, and 240°/s.

Simple linear regression results showed that the peak torque of the concentric knee extensors in the dominant leg (*R*^2^ = 0.513, F = 16.834, *p* < 0.001; R² = 0.372, F = 9.488, *p* < 0.05; *R*^2^ = 0.925, F = 196.714, *p* < 0.001) explained 51.3%, 37.2%, and 92.5% of the variance in CMJ height at all angular velocities, respectively. At all angular velocities, the peak torque of the concentric knee extensors in the non-dominant leg (*R*^2^ = 0.533, F = 18.255, *p* < 0.001; *R*^2^ = 0.806, F = 66.635, *p* < 0.001; *R*^2^ = 0.918, F = 178.976, *p* < 0.001) explained 53.3%, 80.6%, and 91.8% of the variance in CMJ height, respectively. At an angular velocity of 60°/s (Table 5), limb asymmetry in the concentric knee flexors accounted for 22.5% of the variance in CMJ height (*R*^2^ = 0.225, F = 4.653, *p* < 0.05). At the three angular velocities, limb asymmetry in the concentric knee extensors accounted for 31.2%, 40.7%, and 39.1% of the variance in CMJ height, respectively (*R*^2^ = 0.312, F = 7.270, *p* < 0.05; *R*^2^ = 0.407, F = 11.003, *p* < 0.05; *R*^2^ = 0.391, F = 10.284, *p* < 0.05).

**Table 4:** Relationships between the height of the countermovement jump and relative peak torque of the dominant and non-dominant knee extensors across angular velocities.

Velocity	*r*	*p*-value
RPTextensorD60	0.716	<0.001
RPTextensorD180	0.610	0.007
RPTextensorD240	0.962	<0.001
RPTextensorND60	0.730	<0.001
RPTextensorND180	0.898	<0.001
RPTextensorND240	0.958	<0.001

Statistically significant associations *p* < 0.05; RPT, relative peak torque; D, dominant; ND, non-dominant.

**Table 5:** Relationships between the height of the countermovement jump and limb asymmetry across angular velocities.

Velocity	*r*	*p*-value
Concentric Limb Asymmetry Flexor60	−0.475	0.047
Concentric Limb Asymmetry Flexor180	−0.217	0.387
Concentric Limb Asymmetry Flexor240	−0.224	0.371
Concentric Limb Asymmetry Extensor60	−0.559	0.016
Concentric Limb Asymmetry Extensor180	−0.638	0.004
Concentric Limb Asymmetry Extensor240	−0.626	0.005
Eccentric Limb Asymmetry Flexor60	0.184	0.466
Eccentric Limb Asymmetry Flexor180	0.151	0.459
Eccentric Limb Asymmetry Flexor240	0.304	0.220
Eccentric Limb Asymmetry Extensor60	−0.262	0.293
Eccentric Limb Asymmetry Extensor180	−0.262	0.294
Eccentric Limb Asymmetry Extensor240	−0.357	0.146

## Discussion

This study examined the peak torque of knee flexors and extensors among elite gymnasts, as well as the correlation between knee extensor strength and limb asymmetry in relation to CMJ height. Results demonstrated that the peak torque of knee flexors and extensors significantly decreased as the angular velocity increased from 60°/s to 240°/s, whether eccentric or concentric. However, the functional H_ecc_/Q_con_ ratio significantly increased as the velocity increased from 60°/s to 240°/s. The concentric knee extensors exhibited limb asymmetry of 10.02% at an angular velocity of 180°/s. The strongest correlation between knee extensor strength and CMJ performance was observed at 240°/s, and a significant negative correlation was also found between the limb asymmetry and CMJ height.

The differences in the strength of limbs in the knee joint are recognized as risk factors for muscle injury (37). Previous studies indicate that limb strength differences within 10%, 10%–15%, and above 15% are normal, abnormal, and high risk, respectively (38, 39). Though as mentioned in a previous section, the applicability and relevance of said thresholds are somewhat disputed. Nevertheless, results in the present study showed that gymnasts exhibited a concentric extensor limb asymmetry exceeding 10.02% only at an angular velocity of 180°/s. This suggests that gymnasts maintain a balanced bilateral knee muscle strength at most angular velocities but face a risk of asymmetrical extensor muscle strength at a medium speed of 180°/s. Echoing the findings of Sarabon et al. (40), this study reveals that other sports can result in muscle strength imbalances. For instance, Schons et al. (15) research found that volleyball players also exhibited a muscle strength imbalance of 10%–20% in their lower limbs. Bell et al. (22) investigated 167 Division I athletes and revealed that approximately 95% of them had lower limb strength asymmetry ranging from −11.8% to 16.8%. This suggests that limb asymmetry is prevalent in sports. Notably, the strength differences between limbs are influenced by exercise patterns and years of training (41). Elite gymnasts frequently perform vertical somersaults (around the vertical axis) for extended periods, and difficult vertical somersaults require them to adjust their center of gravity to one side of their body at the beginning of the jump to complete the maneuver. This movement pattern falls within the critical power range that requires strength and speed, which may explain the imbalance in muscle strength between limbs. However, this asymmetry is not beneficial. It has been reported that during the propulsion phase of CMJ, the dominant limb applies greater force, compensating for the weaker limb to maintain performance (42). Over the long term, this could potentially lead to injury in the unilateral limb.

Furthermore, this study also found a significant negative correlation between the limb asymmetry and CMJ height at all three angular velocities, which aligns with previous research. Bishop et al. (43) reported a moderate negative correlation between inter-limb asymmetry and vertical jump performance (*r* = −0.47 to −0.59). Of particular note, regression analysis revealed that the limb asymmetry accounted for 40.7% of the variance in CMJ at an angular velocity of 180°. This means that greater inter-limb differences among gymnasts may lead to a decrease in jumping height. While the existing literature presents controversy regarding the impact of inter-limb asymmetry on athletic performance, with some studies indicating task-specificity of its influence (18) and others finding no significant association between limb asymmetry and performance (15, 25, 44). However, the findings of this study provide new evidence to this discussion, particularly within gymnastics, a complex sport highly dependent on symmetry and balance in movements. Gymnastics events, whether involving longitudinal or transverse axis somersaults, require two-footed take-offs and landings (45), which consequently places exceptionally high demands on inter-limb strength balance. Therefore, when coaches develop training plans aimed at enhancing jumping performance, they must thoroughly consider the assessment and intervention of contralateral strength deficits.

When measuring the relationship between concentric knee extensor peak torque and CMJ height across three angular velocities, a significant positive correlation was observed. This indicates that the concentric knee extensor strength plays a key role in the performance of explosive jumps. In gymnastics, the ability to quickly generate peak torque is critical for the propulsion needed to complete high-difficulty aerial movements (46, 47). The results of this study showed significant correlations at 60°/s and 180°/s, with the strongest correlation between the peak torque of the concentric knee extensor and CMJ height being observed at 240°/s. This is consistent with Markovic & Jaric (47), who highlighted the important contribution of knee extensor strength to jumping and sprinting performance, which is consistent with the characteristics of gymnastics (48, 49). However, we also found that at 240°/s, the coefficient of variation (CV%) for concentric knee extensor torque in both dominant and non-dominant legs exceeded 20%, indicating considerable individual variability among gymnasts in high-speed strength output. For coaches, this underscores the importance of individualized training programs that address each athlete's unique strength profile to optimize performance outcomes. Additionally, the regression analysis explained 92.5% of the CMJ at an angular velocity of 240°. In other words, a deficit in knee extensor muscle strength at an angular velocity of 240°/s has the strongest impact on CMJ height. Bradshaw (10) pointed out that sprinting ability and taking off at high speed from the board are fundamental for performing high-difficulty vaults. A similar logic may also apply to floor exercises, such as the explosive take-off during tumbling and short sprints (50). The muscles and tendons of the knee joint possess an excellent elastic function, and strong muscle strength can significantly improve the storage and release of elastic energy (51). Therefore, we suggest that explosive force assessment for the concentric knee extensors be conducted at an angular velocity of 240°/s.

The complexity and high intensity of movements in gymnastics place significant pressure on the knee joint especially during jumping and landing, where the knee joint must absorb the impact from the ground (52). Studies have shown that a functional H_ecc_/Q_con_ ratio of the knee joint not lower than 100% is ideal (53). However, this ratio varies across populations and angular velocities. Results showed that the functional H_ecc_/Q_con_ ratio of Chinese gymnasts was around 87% at a low speed of 60°/s, and between 0.98% and 1.26% at a medium speed of 180°/s and a high speed of 240°/s, all of which aligned closely with the findings of previous research. Lee et al. (28) found that the H_ecc_/Q_con_ ratio of soccer players during high-speed movements typically exceeded 100% at medium to high angular velocities. A higher H/Q ratio provides effective stabilization and shock absorption during jumping and landing, thus reducing anterior shear forces on the tibia and the risk of anterior cruciate ligament injury (54). For comparison, at low angular velocities, the functional H/Q ratio for soccer and basketball players is typically less than 100% (8, 55). In sum, the H/Q ratio consistently increases as the angular velocity increases for various tasks (5). This may be attributed to the contribution of the muscle's elastic components, as faster muscle contractions lead to a decrease in the peak torque of concentric extensors, while the eccentric peak torque of the flexors appears to remain unchanged (7). Additionally, the H/Q ratio at medium and high speeds aligns with the characteristics of high-speed and explosive gymnastics events. Long-term high-intensity training results in the differing functions of the flexor and extensor muscles at various angular velocities, which subsequently lead to muscle adaptability (56).

Although this study produced important findings, some limitations warrant further exploration. First, the study was confined to 18 elite male gymnasts, which restricts the generalizability of the findings and the statistical power. While *post-hoc* power analysis indicated a statistical power of 0.95, the results should still be interpreted cautiously given the limited sample size. Second, no force platform was used, which may limit the discoveries when measuring explosive task performance. Future studies should further explore the role of jump in gymnastics movements, such as power development, and include samples of different levels and genders to obtain highly comprehensive findings.

## Conclusion

This study revealed that elite Chinese gymnasts exhibited an abnormal limb asymmetry in concentric knee extensors at an angular velocity of 180°/s, indicating a potential risk of injury. Notably, the concentric extensor limb asymmetry at 180°/s showed the strongest significant negative correlation with CMJ height. Furthermore, knee extensor strength demonstrated a significant positive correlation with CMJ height across different angular velocities, with the strongest correlation observed at 240°/s. Based on these findings, we recommend that gymnasts undergo regular isokinetic strength assessments, with a particular focus on concentric knee extensor strength and limb asymmetry, as they exhibit moderate to high correlations with CMJ height.

## Data Availability

The original contributions presented in the study are included in the article, further inquiries can be directed to the corresponding author.
